# The quantum mitochondrion and optimal health

**DOI:** 10.1042/BST20160096

**Published:** 2016-08-15

**Authors:** Alistair V.W. Nunn, Geoffrey W. Guy, Jimmy D. Bell

**Affiliations:** * Research Centre for Optimal Health, Department of Life Sciences, University of Westminster, London W1W 6UW, U.K.; † GW Pharmaceuticals, Porton Down, Salisbury, Wiltshire SP4 0JQ, U.K.

**Keywords:** aging, cognition, hormesis, inflammation, mitochondria, quantum, thermodynamics

## Abstract

A sufficiently complex set of molecules, if subject to perturbation, will self-organize and show emergent behaviour. If such a system can take on information it will become subject to natural selection. This could explain how self-replicating molecules evolved into life and how intelligence arose. A pivotal step in this evolutionary process was of course the emergence of the eukaryote and the advent of the mitochondrion, which both enhanced energy production per cell and increased the ability to process, store and utilize information. Recent research suggest that from its inception life embraced quantum effects such as ‘tunnelling’ and ‘coherence’ while competition and stressful conditions provided a constant driver for natural selection. We believe that the biphasic adaptive response to stress described by hormesis–a process that captures information to enable adaptability, is central to this whole process. Critically, hormesis could improve mitochondrial quantum efficiency, improving the ATP/ROS ratio, whereas inflammation, which is tightly associated with the aging process, might do the opposite. This all suggests that to achieve optimal health and healthy aging, one has to sufficiently stress the system to ensure peak mitochondrial function, which itself could reflect selection of optimum efficiency at the quantum level.

## Introduction

In the light of recent scientific advances Theodosius Dobhanzky's quote should be updated [1]: nothing in biology makes sense except in the light of evolution *and quantum physics*. It is now proposed that evolution and natural selection of self-replicating molecules started, in a chemical sense, well before recognisable biological life developed [2]. From a thermodynamic perspective, life can be described as a ‘dissipative structure’ driven by an energy gradient that increases the entropy of its surroundings. Despite the energy cost of storing and utilizing information, natural selection selects the fittest. In effect, some molecules, if subject to perturbation, appear to self-organize and show emergent behaviour leading to complexity [3].

This flow of information is dependent on electric fields and can take the form of just about any type of molecule–ranging from electrons and protons, to metal ions, neurotransmitters, hormones, proteins, energy molecules, RNA and DNA. Environmental perturbation generates a signal that initiates a corrective response. Systems that fail to do this are rapidly eliminated by natural selection. A cell can thus be viewed thermodynamically as a semi-open system that allows energy to enter and waste entropy to leave. As a cell grows, it becomes more and more difficult for it to maintain internal order due to a rapid increase in volume, but as soon as it undergoes mitosis, the smaller daughter cells increase their ‘order’. In effect, basic thermodynamic effects drive replication. Similarly, if energy levels fall, it becomes increasingly difficult for the cell to survive as its internal order also falls, ultimately leading to death [4]. This therefore is just another way of saying that living beings ‘eat order and excrete negative entropy’ [5].

From a biological point of view the ability to process and utilize information could be described as ‘intelligence’. This would suggest that mild stresses that perturb homoeostasis resulting in beneficial adaptation to better resist it, otherwise known as hormesis, may underlie the evolution of intelligence–and could play a role in maintaining it [6]. In effect, all life displays ‘intelligence’ as an inevitable consequence of natural selection in a variable environment. The flip side to this is of course that life, and therefore intelligence, could not have evolved in a totally benign environment, and that reduction or removal of hormetic stresses would lead to a slow deviation from optimal function. Given that ‘quantum effects’ are being recognized as pivotal in many fundamental biological processes [7], it follows that together with the concept of ‘hormesis’, the ability to live in ‘optimal health’ must encompass these principles.

‘Hormesis’ describes a biological phenomenon that has long been observed whereby a low dose ‘stressor’ induces adaptation in an organism such that it can better resist it. It was only given a name in the 1940s from the ancient Greek ‘hormáein’, meaning ‘to urge on’ [8]. The concept has had a chequered history and it is only recently becoming more widely accepted within the scientific and clinical community [9]. In biological terms hormesis has been described as ‘an organismal strategy for optimal resource allocation that ensures homoeostasis is maintained’ [10]. Classically, hormesis has also been described by toxicologists as a biphasic response of low dose stimulation and/or beneficial effect and high dose inhibitory and/or toxic effect; it has also been defined by Mattson as ‘a process in which exposure to a low dose of a chemical agent or environmental factor that is damaging at higher doses induces an adaptive beneficial effect on the cell or organism’ [11]. Today we know that multiple mechanisms underpin hormesis [12] and that it has been widely observed in the toxicological literature; these data are summarized in a database (see [13]). Overall, because hormesis can often induce many benefits biologically, it may be essential in disease prevention and possibly treatment.

## From thermal vents to advanced intelligence; the mitochondrion

One of the strongest emerging theories, due to the ubiquity of the proton gradient in cells, is that life commenced in alkaline thermal vents at the bottom of the oceans. These vents exhibit large stable proton gradients which over 1000s of years, and with natural selection, probably gave rise to two orders of life–archaea and bacteria. At some point these two forms came together in a biosymbiotic coupling, with the latter becoming the mitochondria, which enabled the development of the eukaryote and complex life. Critically, because most of the bacterium's genes ended up in the nucleus, and only a few stayed in the nascent mitochondrion, this forced the evolution of two sexes due to the need to ensure minimal mitochondrial heteroplasmy. Any large mismatch between mitochondrial genes encoded in the nucleus and those encoded in the mitochondrion could result in reduced efficiency of the electron transport chain (ETC), so potentially enhancing ROS and reducing energy production; it is thought that this could determine both lifespan and the rate of aging [14].

Thus mitochondria have been key in the evolution of complex life, as they enable vast amounts of ‘information’ to be stored and processed in a cell by supplying almost unlimited amounts of energy [15]. Apart from the importance of DNA transferring information between generations, from the living organism's perspective, it is also important to ‘remember’ the past, ‘predict’ the future and therefore be ‘aware’ of the present. It therefore follows that memory has been defined as the capacity of organisms to benefit from their past [16]. Without energy there can be no memory, and thus no awareness. This may explain why our brains require so much; even at rest approximately 20% of the total body energy consumption arises from the brain. Stimulate it, and it rapidly increases its energy demand, however, the precise increase in energy use by action potentials from baseline in the resting states is thought to be only approximately 10% or so, with the rest of the energy being used on housekeeping tasks, resting potential, postsynaptic receptors, neurotransmitter recycling, vesical cycling and calcium homoeostasis [17]. The majority of the energy is supplied by mitochondria and is consumed at the synapses [18]. Calculations suggest that the active brain can generate approximately 30 μmol ATP/g·min, which is not too dissimilar to what a human leg muscle is generating during a marathon [19]. In contrast, anaesthesia reduces cerebral baseline metabolic rate by 30–70% [20]. Although sleep is also associated with decreased metabolism, it is essential to restore optimum performance [21]; bigger brains may have evolved, in part, to decrease the need for sleep by reducing the time taken to clear metabolites by decreasing the neuronal density to area ratio [22]. Thus the mitochondrion is essential for advanced informational structures like the brain. Certainly in humans, research does suggest that fluid intelligence is related to the metabolic efficiency of the mitochondrion [23].

## Stress is required to maintain the complexity of the brain

The energy requirements of the brain are not surprising, given its complexity. The human brain has approximately 80–100 billion neurons, approximately a 10-fold increase since our Miocene ancestors 10 million years ago [24,25]. In humans, one estimate suggests that there could be an average of approximately 7000 synapses per cell in the neocortex, with a total of approximately 0.15×10^15^ synapses in the cortex [26]. Others have suggested that there may be as many as 10^14^ synapses in the cerebral cortex, and approximately 10^13^ in the cerebellar cortex [27]. Overall, this indicates that the human brain contains at least 10^14^ to 10^15^ synapses. Furthermore, there may be at least 26 distinguishable synaptic strengths, corresponding to 4.7 bits of information at each synapse [28]. As a byte is generally considered to contain eight bits, this might suggest that the brain could hold between 0.58×10^14^ and 0.58×10^15^ bytes of information or between 58 and 580 terabytes.

The brain can therefore be viewed as an immensely complex structure that has evolved in response to the need to adapt and take on more information–and is fundamentally an information-collecting dissipative structure driven by environmental challenge. And as many studies show, not stimulating it, either through direct use, say of cognitive tasks, or via performing complex movements during physical activity, its performance tends to drop. It therefore echoes the thermodynamic rules underling the very beginnings of life; perturbation of a complex set of molecules can induce order–take the stress away and its structure begins to unravel [29]. But perhaps one of the brain's most interesting aspects is that it can also be analysed in relation to free energy and information theory; the human brain is vastly more efficient than current electronic devices, and uses multiple mechanisms to do this, including miniaturization [30].

## The quantum angle

The brain is not simply a computer system and as Roger Penrose has proposed, it may utilize quantum principles to enable it to process information and generate awareness [31]. Quantum theories of the mind have led to a whole new field of science–‘quantum neurophysics’ [32], which mirrors the idea that life is anchored in the quantum world [33]. Interestingly, it is now becoming clear that bacteria can transfer electrons both between the same species and with other species in a form of symbiosis via ‘bacterial nanowires’. In effect, these are biological conductors; they can transfer energy. Significantly, this ‘conductance’ appears to have been solved in at least two ways by nature: one is more similar to classical metallic conductance based on free electron theory, whereas the other seems to depend on quantum effects–such as ‘tunnelling’ [34]. It therefore seems likely that the brain is probably also using quantum effects at some level–although precisely how much, or how little, is still unknown.

Quantum literally means ‘how much’, but is today used to describe the minimum unit of energy or matter. It was Planck who realized that there was a minimal ‘*quantum of action*’, in effect, there is a minimum change that can be measured in nature, which became known as Planck's constant, or *h*, which equals 6.6×10^−34^ J/s. The implications from this were profound, not least of which were that any measurement of nature is based on quantum effects, and that the size and shape of things is also determined by Planck's constant. It also means that there is always motion within matter; at the molecular level, the shape of things is determined by an average and motion is therefore ‘fuzzy’, and it is impossible to assign both momentum and position of a particle. It also means that the so called ‘energy barriers’ normally encountered in most physical/biological/chemical system may not be barriers at all. This describes one of the most fascinating principles of the quantum world, ‘tunnelling’. The phenomenon of ‘tunnelling’ explains how objects can permeate energy barriers without the necessary energy because they can exist as probability waves; the likelihood of this can be predicted by the Schrödinger equation. This basically tells us that the ability to do this depends on their energy and mass, and the width of the barrier. It is actually quite likely for very small particles like electrons and protons, but extremely unlikely for large objects such as humans. Thus increasing temperature can enhance the effect as it can impart more energy, although as we will discuss later, it also can inhibit it. The possibility that electrons could move along enzymes in such way was first suggested by Szent-Györgyi in 1941 [35], but it was DeVault and Chance in 1966 [36] who proposed it could be due to quantum tunnelling. See Box 1 for a more in depth explanation of some aspects of quantum physics.

Box 1. **A bit more on quantum physics**

To explain quantum tunnelling, one of the basic concepts underlying the quantum world is that of wave-particle duality; De Broglie showed that just as a photon can behave both as a wave and a particle, all particles could have a ‘wave function’ ascribed to them–matter-waves. This was pivotal, as electrons could thus also behave as waves. The wave function also displays something called ‘phase’, in effect quantum particles behave as a rotating cloud, and thus can be influenced by magnetic fields; they have ‘spin’. Spin explains Pauli's exclusion principle and why atoms, or planets, don't collapse in on themselves and matter feels ‘hard’. However, tunnelling also depends on ‘quantum coherence’ such that an electron, proton, atom or a group of atoms, exist in ‘quantum superposition’–in effect, it or they exist as a collection of all possible states. Another facet of this is ‘entanglement’, or as Einstein put it, ‘spooky action at a distance’–which describes the ability of two entangled particles to ‘know’ the state of the other when one is observed, regardless of distance–instantaneously. This is known as ‘non-locality’, as encompassed by Bell's theorem; this is a profound departure from classical physics. Bell's inequality has now been tested repeatedly, and the most recent experiment does strongly suggest that quantum entanglement is entirely real [37]. From the quantum point of view, once entangled, two particles have to be regarded as the same entity, irrespective of distance. Thus entanglement is not only key to understanding reality, but is key to many current and future technologies, including quantum computing [38].

However, when particles are observed, they appear in one particular state and thus display classical properties we associate with the everyday world. Thus to exist in a non-classical quantum state, they need to be isolated from external interference from the environment; as soon as this system interacts with it, it becomes ‘*decoherent*’ and they would appear to behave as particles rather than probability waves; effectively they are being ‘*observed*’ (a sort of Schrödinger's cat condition). The more particles involved, the quicker the quantum state collapses–as maintaining a coherent state becomes increasingly difficult with increasing size due to interaction with the environment. This is why a tennis ball, although it can be technically be assigned a wave length, is always observed as a tennis ball; calculating its de Broglie wavelength, which is obtained by dividing the Planck constant by the ball's momentum, is approximately 10^−34^ m, whereas that of an electron, with a rest mass energy of 0.511 MeV, at 1 eV, is 1.2 nm. It is also why a cat does not exist in superposition; it is intimately coupled to its environment. The important message here is that microscopically, coherence is possible, not only for single entities, such as electrons, but also for larger groups of atoms–indicating that they can behave as one entity. But this state is rapidly lost via interaction with the wider environment; this is explainable thermodynamically, because most ‘environments’ contain vast number of molecules that display randomness. This is why we view the world macroscopically. For a basic introduction to quantum physics, a good starting point is the 30-second quantum theory book, edited by Brian Clegg [39], or for a more detailed over-view, the free to down load text book: ‘Motion mountain–adventures in physics, volume IV, the quantum of change’, edition 28.1, 2016, by Christoph Schiller (http://www.motionmountain.net/) is a good, but more in-depth reference.

It is becoming clear that components of living systems use quantum principles, for instance, by absorbing light energy and transferring it across a series of molecules–a fundamental quantum process, where quantum entanglement can be viewed as a form of quantum superposition [40]. One of the factors that can influence coherence is the environmental temperature, as this indicates the energy of a particle, and thus its ability to interact with other components. The higher its energy, the more likely it is to disrupt it. For many years, it was thus thought that life was simply too ‘*warm and wet*’ for coherence to occur. As it now turns out, this is far from being correct, as life has actually tuned itself to use thermal vibrations to ‘*pump*’ coherence, rather than disrupt it, which results in a phenomenon known as ‘*quantum beating*’. This effect has been detected in bacterial light harvesting complexes and essentially represents a coherent superposition of electronic states, analogous to a nuclear wavepacket in the vibrational regimen. In essence, the energy in light can be harvested very efficiently and transferred using wavelike resonance. There is thus a ‘goldilocks zone’ to optimize efficiency; in effect, just the right amount of ‘noise’ can result in enhanced ‘coherence’ and ‘tunnelling’ due to stimulating particular vibrational modes in proteins–so called exciton-vibrational coupling (vibronic coupling) [41–44]. For example, tubulin contains chromophoric aromatics molecules such as tryptophan, and thus may play a role in coherent energy transfer; key in this maybe their free pi electrons [45,46]. Today long-range electron tunnelling is thought to occur in many proteins, and seems to be enhanced by particular molecules, such as the aromatics–which occur more frequently in oxidoreductases, which are key components of respiratory chains [47]. Crucially, it is now thought that electron tunnelling plays an important role in how mitochondria produce energy [48,49] and ensures a tight coupling between electron flow and protonation via a process known as ‘redox tuning’ [50]. In effect, electron tunnelling appears to be a pivotal component of mitochondrial function. It would be interesting to speculate on the role of temperature in this quantum effect: could temperature increase the ability to tunnel further, but disrupt coherence more readily? Is there a so called ‘sweet spot’?

Certainly, it seems likely that quantum tunnelling and entanglement were essential for the beginnings of life, especially in relation to photosynthesis, allowing a greater spectrum of photons to be gathered and more efficient transfer of electrons [40,51]. Indeed, many biomolecules may have been selected for their ‘quantum criticality’, and thus behave somewhere between an insulator and a conductor, so also potentially acting as charge carriers [52]. Practical examples include quantum effects used in bird navigation [53], an explanation of how photosynthesis works [41], and possibly, even olfaction [54]. The recent discovery that lysozyme appears to demonstrate a ‘Fröhlich condensate’ [55], when combined with concept that strong electro-magnetic fields generated by mitochondria could generate ‘water order’, and thus protect against *decoherence* [56], is perhaps further evidence. In fact, emerging mathematical models suggest that quantum coherence can be maintained for significant periods of time, orders of magnitude longer in complex biological systems than in simple quantum systems at room temperature–in effect the system can hover in the ‘Poised Realm’ between the pure quantum and incoherent classical worlds [57]. Thus, although computers may rely on quantum principles, life has been using them since the beginning, and what we see today is the result of billions of years of natural selection. So it appears that to fully understand biology, we have to embrace the quantum world, and this may begin to explain why life is generally so efficient.

## The quantum mitochondrion

Clearly a lot more ‘quantum effects’ are taking place in mitochondria than previously assumed. Certainly, the close association between ROS generation and the ETC, and the discovery of ‘mitochondrial oscillators’, which has enhanced the understanding of complex non-linear systems [58]–is highly relevant. Data suggest that mitochondria have evolved to generate energy at a ‘redox sweet spot’, where without too much stress, they can maximize energy production with minimal ROS, but if the ETC becomes either too reduced or oxidized, ROS signalling occurs–the so called ‘Redox-Optimized ROS Balance’ (R-ORB) hypothesis; a key component of this is antioxidant defence [59]. The combination of increased ROS and increased ADP/ATP is a powerful signal for mitochondrial biogenesis and/or localized induction of production of ETC components. The latter effect is well described by the CoRR hypothesis (Colocation of gene and gene product for Redox Regulation of gene expression) [60]. In this instance, this would have a number of effects ranging from stimulation of growth, to a localized activation of uncoupling proteins (UCPs), which are activated by ROS; these are well described effects relating to redox [61].

If electron tunnelling is so important in controlling electron flow through the ETC, does this indicate that other quantum effects may also be involved? Could ‘entanglement’ be used to signal? For instance, during electron bifurcation, it has been suggested that the semiquinone-Rieske cluster can exist in a triplet state in complex III involving a spin–spin exchange; during this reaction, two electrons are taken from ubiquinol and sent in two different directions [62]. Interestingly, Marais and colleagues have proposed that as weak magnetic fields can reduce triplet products in photosynthetic organisms, a high-spin Fe^2+^ ion within the ETC can generate an effective magnetic field that can reduce ROS production. In effect, a quantum protective mechanism in photosynthesis [63]. If the triplet state can be used for bird navigation [53], could this hint that it is used in other biological processes as well? The link between triplet states and fields is particularly interesting–suggesting that ROS could be signalling in more ways than we realized.

But quantum effects are not just limited to electrons–*proton tunnelling* may be key in enzymatic reactions [64], whereas other small molecules can also be described by wave functions, for instance, calcium, sodium and potassium. This might mean that these highly important elements, for instance, in enabling action potentials, may also incorporate quantum effects, and may play a role ion channel selectivity; these ideas have been used to account for differences between those predicted by the Hodgkin–Huxley equation and what has been observed in neural circuits [65,66].

There is also one other area that field strength might modulate–and that is mitochondrial dynamics. Skulachev has suggested that fused mitochondria could act as ‘power cables’ [67]; it is thus interesting that Reynaud has shown that mitochondria can be made to fuse using electric fields *in vitro* [68]. This would be in keeping with the ideas of Fröhlich about energy transfer involving vibronic coupling, in particular, between mitochondria and microtubules [69]. Certainly, it has been long known that electrics fields affect cell function and shape: calcium has a strong effect on the electrical energy transfer and transistor-like properties of microtubules [70]. It has also been suggested that differences in mitochondrial function in cancer alter electric fields, in particular, affecting water order and coherence–which could be involved in the disease process [71]. Cancer is clearly associated with changes in mitochondrial dynamics and ultrastructure [72]. Overall, it seems that mitochondrial fusion induced by mild stress or reduced nutrients tends to enhance oxidative phosphorylation, whereas too much stress, excess nutrients, disease and inflammation, including cancer, induces fragmentation which usually leads to mitophagy and reduced oxidative phosphorylation [73].

**Figure 1:**
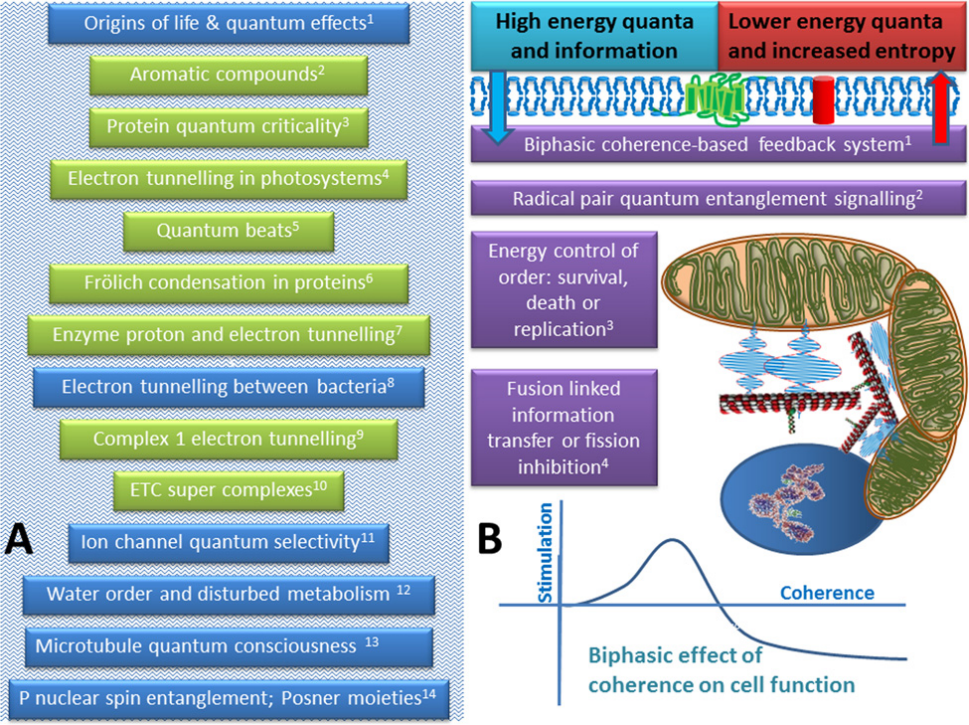
The quantum mitochondrion This figure summarizes some quantum effects in biology: the green boxes represent those that appear to be established, the blue boxes those that have been suggested to be involved and the purple boxes some we believe might be involved. As living systems takes in energy in order to store and utilize information, so effectively using free energy to do work to create a highly ordered state that becomes more efficient and selectable by natural selection, it exports disorder, so maintaining the second law of thermodynamics. Panel (**A**) Proven and predicted quantum effects in biology from the literature: (1) life evolved because of, and has incorporated basic quantum principles, such as entanglement and tunnelling [40,74]; (2) aromatic compounds, such as tryptophan, have pi electrons that can delocalize, and can enable quantum effects [75]; (3) the first real evidence for quantum effects has come from the reliance of photosynthesis on electron tunnelling [41,42]; (4) natural selection appears to have resulted in macromolecules tuned for quantum effects suggesting universal mechanisms of charge transport in living matter [52,57]; (5) quantum beating has been detected in living systems, in particular, in photosystems, suggesting life is using quantum effects [41,43,46]; (6) over 40 years ago Fröhlich predicted that vibrational modes within proteins could condense leading to macroscopic coherence, this appears to have now been observed [55]; (7) tunnelling is now thought to be essential in both enzyme reactions and energy transfer, a quintessential component of the quantum world [47–51,64,76]; (8) bacteria live in colonies, often sharing electrons with different species, and it seems that electrons are ‘transported’ over long distances–it would be surprising if tunnelling was not involved, and this sharing between archaea and bacteria is suggestive of this process being adopted in eukaryotes [47,77–79]; (9) several groups now think that electron tunnelling is important in the ETC [48–50]; (10) an important component of electron tunnelling is the existence of super-complexes–these now appear to exist in all orders of life, and are key in both photosynthesis and respiration [80–82]; (11) ion channels play a key role in the brain, and it has been suggested that ion conduction could be described using quantum principles [65,66]; (12) alterations in electric fields surrounding the mitochondrion could play a significant role in changing ‘water order’ and be associated with disease states [56,83,84]; (13) long discussed theory that microtubules could be involved in resonant energy transfer and consciousness due to their quantum properties [45,75]; (14) the nuclear spin properties of phosphorous utilized by transfer of quantum entangled pairs across the synapse in Posner molecules, so effectively acting as a ‘qubit’ [85]. Panel (**B**) Some predictions of our own: (1) coherence could be controlled by mitochondrial potential, which is turn could enhance quantum tunnelling of electrons (as well, as possibly, other coherence-dependent functions, such as enzyme function or ion channel status)–it could be biphasic as it could enhance efficiency (high ATP, low ROS), but if too high, it could start to hinder ATP by producing too much ROS. Thermodynamically it could initially aid in survival of the individual cell, then favour replication, and if necessary, induce cell death–thus would be hormetic; (2) it is now thought that a radical pair mechanism could be involved in avian navigation (effectively unpaired quantum entangled electron) [53], which might suggest that if such entities were produced in the ETC, it could act as a signal–implying there is more to ROS than thought; (3) if we combine (2) and (3), then any shift in electron flow and/or proton flow, either via electron input, or use, in particular, availability of oxygen, or even cell shape, or damage, would rapidly generate a signal that could instantaneously control mitochondrial function; (4) the effect of mitochondrial dynamics would thus have to viewed in a new light, as it is possible that fusion could enhance a quantum-based signalling system throughout the entire cell, which would be altered by fission.

This might suggest a quantum control system. For example, too little energy production coupled to increased usage is indicated by an increase in the ADP/ATP ratio, which would be associated with increased ETC oxidation, which might initially reduce ROS, but the collapsing mitochondrial membrane potential (mΔΨ) might reduce quantum coherence. This might reduce quantum tunnelling efficiency, which could then lead to an increase in ROS. Hence, both calorie restriction and increased metabolic demand would generate an adaptive response (hormetic trigger) to improve mitochondrial function. As the mitochondrial potential is restored, and mitochondrial mass and/or efficiency increased, quantum tunnelling would become more efficient and ATP levels re-established and ROS minimized. Equally, if the cell is exposed to high levels of nutrients, but does not use much ATP, then the ETC would become highly reduced and the mitochondrion could hyperpolarise–in the presence of oxygen this might lead to a rapid flow of electrons through the ETC and the formation of free radicals, which also effectively inhibit functioning. This points at a quantum coherence ‘sweet spot’, where the field strength has to be just right. An interesting possibility is that the sweet spot could coincide with a degree of mitochondrial fusion and alignment of the fields. Overall this means it is necessary to think ‘quantum’ when viewing how mitochondria function; Figure 1 summarizes the concept.

## Why stress (hormesis) is needed for optimal health

When all of the above is put together it suggests that natural selection has worked over billions of years to incorporate all possible quantum efficiencies in response to stress and is based on the emergent behaviour resulting from the perturbation of a complex system. The captured information that enables this is encoded in DNA, which is the result of billions of cycles of informational storage, which might be explained by the informational cycle of Brillouin [86]. This means that although life can keep going without too much stress, it is very likely that its robustness will decrease if it is not perturbed, as a key factor maintaining structure, natural selection of efficient systems under stress, has been removed. However, with the right amount of stress, the most efficient system is maintained. A key marker for this is mitochondrial health, which plays a fundamental role in the aging process.

Recent published literature suggest that the human lifespan may be fixed, possibly at a maximum of approximately 125 years–with an asymptotic limit approximately 95 years [87–89]. However, the rate of aging is modifiable leading to the current situation of ‘accelerated aging’ in an obesogenic environment [90]; key to this process is its association with rising inflammation [91–93]. On the other hand it opens up the possibility for ‘healthy aging’. It has been long assumed that calorie restriction, which seems to suppress reproduction and increase longevity, improves somatic maintenance and suppresses excessive inflammation–possibly through resource reallocation [94]. However, it has also been suggested that it may not be simply about somatic maintenance per se, rather, calorie restriction-induced slowing of aging is simply a secondary effect brought about by increased autophagy and apoptosis to divert resources to support reproduction [95]. Further data that the lifespan is fixed, but with a modifiable asymptotic span, comes from the discovery of the epigenetic clock [96,97]. Thus although aging does have a stochastic element, there is a good argument that it is programmed, and this is related to epigenetic control of development–and is an example of antagonistic pleiotropy, the so called ‘short-sighted’ watchmaker hypothesis [98]. Some insight into this comes from the mitochondrion, which quite apart from controlling death, also controls the epigenome through Krebs's cycle intermediates [99,100], which is itself controlled by inflammation that can increase ROS production [101]. Interestingly, the longer lived a species is, the more efficient its ETC, which results in a lower production of ROS, requiring less investment in antioxidant mechanisms and DNA repair [102]. The key point here is that mitochondrial function does decline with age and seems to be related to a Muller's ratchet mechanism amplifying damaging mitochondrial DNA mutations–and is matched by a down-regulation of genes involved in mitochondrial function, but an up-regulation of innate immune genes [103]. Evidence does suggest a definite increase in somatic mtDNA heteroplasmy with age [104]. This of course suggests a very tight relationship between mitochondrial function, quantum efficiency, inflammation and aging.

What is clear is that although the global average life expectancy has increased in the last 20 years, the relative ‘*healthy life expectancy*’ (HALE) has not kept pace. For instance, in the UK, from 1990 to 2010, male HALE at birth rose from 62.8 to 65.7 and female from 65.9 to 67.9 years. In comparison, the absolute male life expectancy rose from 72.9 to 77.8 and the female from 78.3 to 81.9 years respectively. In effect, although absolute life expectancy has increased by 6.3 and 4.4% in males and females, respectively, HALE only rose by 4.5 and 3%, respectively, over the same time period. This is reflected globally, and seems to be mainly due to reductions in child and adult mortality, rather than years lost to disease–suggesting ‘*morbidity expansion*’ [105]. In effect, although average global life expectancy has gone up, it does not seem to be due to an increase in HALE, and is certainly not approaching anything like that possible for a human.

One key driver for morbidity expansion is *lifestyle-induced inflammation* which is known to alter mitochondrial function, leading to accelerated aging. Reducing this might lead to a slowing of the aging rate. The best way to achieve this is no doubt via introduction of hormetic factors, such as exercise, and reduction of inflammation-inducing conditions, such as obesity. It has been long known that mitochondria play a key role in hormesis, as mildly stressing them induces a rebound adaptive response to improve their efficiency [106]. Inflammation, however, evolved to resist pathogens and invoke repair of damage and utilizes mitochondrion to this end–changing their function to increase ROS [101]; this, by its very nature, initially destroys larger structures. Although a good inflammatory response is essential for survival, it can rapidly accelerate the aging process if it becomes chronic. Thus, optimal health should not be viewed simply as an absence of disease, but rather as the induction of a more robust system that can more ably maintain homoeostasis in the face of challenges. In effect, we suggest that hormesis selects for the most efficient ETC, which slows down a perhaps inevitable feed forward loop of inflammation-driven mitochondrial dysfunction. Of course, evolution also selected for the induction of an inefficient ETC during inflammation. In fact, it is now becoming clear that the development of an innate immune system, and programmed cell death is ancient, evolving in prokaryotes [107–109]. So the fine tuning of the ETC to optimize survival of a species is truly ancient, and is very likely to encompass basic quantum effects. For modern warm blooded animals, this could even include temperature itself.

The inter-relationship and boundary between the coherent microscopic quantum realm, and the essentially decoherent macroscopic one could therefore be telling us a great deal. For example, the imposition of the decoherent environment on to a coherent one inside the mitochondrion would immediately change its state. It could be argued that this is in effect, hormetic. Induction of temporary decoherence in a system that normally relies on coherence, such at the ETC, would be a trigger to enhance adaptive function–for example, by creation of ROS. Thus aspects of mitochondrial function could be operating at the boundary between the quantum and classical world, where the environment modulates the. This might suggest that the mitochondrion could be acting as a sensor balanced between the two realms. Any change instantaneously alters its output. It is thus the interplay between the macroscopic world of decoherence and the microscopic world of coherence that determines mitochondrial function.

In summary, the central precept for this paper is that humans evolved in a quantum universe and that quantum effects are pivotal for optimal function. Central to this is that the emergence of complex systems can only take place in the presence of perturbation, where evolution selected for the ability to take on and process information. In effect, life and intelligence could be said to be one and the same thing. With the emergence of life came competition, which coupled with environmental challenges and the relentless imposition of natural selection, led to the evolution of higher and higher orders of intelligence and cognitive capabilities. Pivotal to this whole process was hormesis and its impact on mitochondria. Of course, a key survival strategy, besides adaptation, is the use of information to alter the environment, so providing a competitive edge. Humans reached this point 1000s of year ago suggesting that we should be living in good health throughout adult life. However, it seems that by making ourselves too comfortable and removing hormetic stressors, we are not achieving optimal mitochondrial quantum efficiency and thus are unable to achieve and maintain optimal health.

## Abbreviations

ETC: electron transport chain
HALE: healthy life expectancy

## References

[1] Dobzhansky T. (1973). Nothing in Biology Makes Sense except in the Light of Evolution. Am. Biol. Teacher.

[2] Pross A. (2012). What is Life? How Chemistry Becomes Biology.

[3] Laughlin S.B., de Ruyter van Steveninck R.R., Anderson J.C. (1998). The metabolic cost of neural information. Nat. Neurosci..

[4] Gatenby R.A., Frieden B.R. (2013). The critical roles of information and nonequilibrium thermodynamics in evolution of living systems. Bull. Math. Biol..

[5] Schrodinger E. (1944). What is Life?. The Physical Aspect of the Living Cell.

[6] Nunn A.V., Guy G.W., Bell J.D. (2014). The intelligence paradox; will ET get the metabolic syndrome? Lessons from and for Earth. Nutr. Metab..

[7] Arndt M., Juffmann T., Vedral V. (2009). Quantum physics meets biology. HFSP J.

[8] Southam C.M.E.J. (1943). Effects of extract of western red-cedar heartwood on certain wood-decaying fungi in culture. Phytopathology.

[9] Calabrese E.J. (2011). Toxicology rewrites its history and rethinks its future: giving equal focus to both harmful and beneficial effects. Environ. Toxicol. Chem..

[10] Calabrese E.J., Baldwin L.A. (2002). Defining hormesis. Hum. Exp. Toxicol..

[11] Mattson M.P. (2008). Hormesis defined. Ageing Res. Rev..

[12] Calabrese E.J. (2013). Hormetic mechanisms. Crit. Rev. Toxicol..

[13] Calabrese E.J., Blain R.B. (2011). The hormesis database: the occurrence of hormetic dose responses in the toxicological literature. Regul. Toxicol. Pharmacol..

[14] Lane N. (2015). The Vital Question: Why is Life the Way It Is?.

[15] Lane N., Martin W. (2010). The energetics of genome complexity. Nature.

[16] Tulving E. (1985). How many memory systems are there?. Am. Psychol..

[17] Howarth C., Gleeson P., Attwell D. (2012). Updated energy budgets for neural computation in the neocortex and cerebellum. J. Cereb. Blood Flow Metab..

[18] Harris J.J., Jolivet R., Attwell D. (2012). Synaptic energy use and supply. Neuron.

[19] Attwell D., Laughlin S.B. (2001). An energy budget for signaling in the grey matter of the brain. J. Cereb. Blood Flow Metab..

[20] Hudetz A.G. (2012). General anesthesia and human brain connectivity. Brain Connect..

[21] Krueger J.M., Frank M.G., Wisor J.P., Roy S. (2015). Sleep function: toward elucidating an enigma. Sleep Med. Rev..

[22] Herculano-Houzel S. (2015). Decreasing sleep requirement with increasing numbers of neurons as a driver for bigger brains and bodies in mammalian evolution. Proc. Biol. Sci..

[23] Nikolaidis A., Baniqued P.L., Kranz M.B., Scavuzzo C.J., Barbey A.K., Kramer A.F., Larsen R.J. (2016). Multivariate associations of fluid intelligence and NAA. Cereb. Cortex.

[24] Azevedo F.A., Carvalho L.R., Grinberg L.T., Farfel J.M., Ferretti R.E., Leite R.E., Jacob Filho W., Lent R., Herculano-Houzel S. (2009). Equal numbers of neuronal and nonneuronal cells make the human brain an isometrically scaled-up primate brain. J. Comp. Neurol..

[25] Lent R., Azevedo F.A., Andrade-Moraes C.H., Pinto A.V. (2012). How many neurons do you have? Some dogmas of quantitative neuroscience under revision. Eur. J. Neurosci..

[26] Pakkenberg B., Pelvig D., Marner L., Bundgaard M.J., Gundersen H.J., Nyengaard J.R., Regeur L. (2003). Aging and the human neocortex. Exp. Gerontol..

[27] Braitenberg V. (2002). In defense of the cerebellum. Ann. N.Y. Acad. Sci..

[28] Bartol T.M., Bromer C., Kinney J., Chirillo M.A., Bourne J.N., Harris K.M., Sejnowski T.J. (2015). Nanoconnectomic upper bound on the variability of synaptic plasticity. ELife.

[29] Nunn A.V., Guy G.W., Bell J.D., Farooqui T., Farooqui A.A. (2015). Hormesis and cognitive function: an evolutionary/adaptive arabesque leading to longevity. Diet and Exercise in Cognitive Function and Neurological Diseases.

[30] Sengupta B., Friston K.J., Penny W.D. (2014). Efficient gradient computation for dynamical models. NeuroImage.

[31] Penrose R. (1994). Shadows of the Mind; A Search for the Missing Science of Consciousness.

[32] Tarlaci S., Pregnolato M. (2016). Quantum neurophysics: from non-living matter to quantum neurobiology and psychopathology. Int. J. Psychophysiol..

[33] Al-Khalili J., McFadden J. (2014). Life on the Edge: The Coming of Age of Quantum Biology, Transworld Publishers, Great Britain.

[34] Lovley D.R., Malvankar N.S. (2015). Seeing is believing: novel imaging techniques help clarify microbial nanowire structure and function. Environ. Microbiol..

[35] Szent-Gyorgyi A. (1941). Towards a new biochemistry?. Science.

[36] DeVault D., Chance B. (1966). Studies of photosynthesis using a pulsed laser. I. Temperature dependence of cytochrome oxidation rate in chromatium. Evidence for tunneling. Biophys. J..

[37] Schmied R., Bancal J.D., Allard B., Fadel M., Scarani V., Treutlein P., Sangouard N. (2016). Bell correlations in a Bose–Einstein condensate. Science.

[38] Gribbin J. (2013). Computing with Quantum Cats; From Alan Turing to Teleportation.

[39] Ball P., Clegg B., Clifford L., Close F., Hebden S., Hellemans A., Holgate S.A., May A. (2014). 30-Second Quantum Theory; The 50 Most Important Thought-Provoking Quantum Concepts, Each Explain in Half a Minute.

[40] Tamulis A., Grigalavicius M. (2014). Quantum entanglement in photoactive prebiotic systems. Syst. Synth. Biol..

[41] Engel G.S., Calhoun T.R., Read E.L., Ahn T.K., Mancal T., Cheng Y.C., Blankenship R.E., Fleming G.R. (2007). Evidence for wavelike energy transfer through quantum coherence in photosynthetic systems. Nature.

[42] Fassioli F., Dinshaw R., Arpin P.C., Scholes G.D. (2014). Photosynthetic light harvesting: excitons and coherence. J. R. Soc. Interface.

[43] Lim J., Palecek D., Caycedo-Soler F., Lincoln C.N., Prior J., von Berlepsch H., Huelga S.F., Plenio M.B., Zigmantas D., Hauer J. (2015). Vibronic origin of long-lived coherence in an artificial molecular light harvester. Nat. Commun..

[44] Weber S., Ohmes E., Thurnauer M.C., Norris J.R., Kothe G. (1995). Light-generated nuclear quantum beats: a signature of photosynthesis. Proc. Natl. Acad. Sci. U.S.A..

[45] Craddock T.J., Friesen D., Mane J., Hameroff S., Tuszynski J.A. (2014). The feasibility of coherent energy transfer in microtubules. J. R. Soc. Interface.

[46] Craddock T.J., Priel A., Tuszynski J.A. (2014). Keeping time: could quantum beating in microtubules be the basis for the neural synchrony related to consciousness?. J. Integr. Neurosci..

[47] Winkler J.R., Gray H.B. (2014). Long-range electron tunneling. J. Am. Chem. Soc..

[48] Hayashi T., Stuchebrukhov A.A. (2011). Quantum electron tunneling in respiratory complex I. J. Phys Chem. B.

[49] Moser C.C., Farid T.A., Chobot S.E., Dutton P.L. (2006). Electron tunneling chains of mitochondria. Biochim. Biophys. Acta.

[50] de Vries S., Dorner K., Strampraad M.J., Friedrich T. (2015). Electron tunneling rates in respiratory complex I are tuned for efficient energy conversion. Angew Chem. Int. Ed. Engl..

[51] Trixler F. (2013). Quantum tunnelling to the origin and evolution of life. Curr. Org. Chem..

[52] Vattay G., Salahub D., Csabai I., Nassimi A., Kaufmann S.A. (2015). Quantum criticality at the origin of life. J. Phys. Conf. Ser..

[53] Zhang Y., Gennady P.B., Kais S. (2015). The radical pair mechanism and the avian chemical compass: quantum coherence and entanglment. Int. J. Quantum Chem..

[54] Gane S., Georganakis D., Maniati K., Vamvakias M., Ragoussis N., Skoulakis E.M., Turin L. (2013). Molecular vibration-sensing component in human olfaction. PloS One.

[55] Lundholm I.V., Rodilla H., Wahlgren W.Y., Duelli A., Bourenkov G., Vukusic J., Friedman R., Stake J., Scheider T., Katona G. (2015). Terahertz radiation induces non-thermal structural changes associated with Frohlich condensation in a protein crystal. Struct. Dyn..

[56] Pokorny J., Pokorny J., Kobilkova J. (2013). Postulates on electromagnetic activity in biological systems and cancer. Integr. Biol..

[57] Vattay G., Kauffman S., Niiranen S. (2014). Quantum biology on the edge of quantum chaos. PloS One.

[58] Aon M.A., Cortassa S., O'Rourke B. (2008). Mitochondrial oscillations in physiology and pathophysiology. Adv. Exp. Med. Biol..

[59] Cortassa S., O'Rourke B., Aon M.A. (2014). Redox-optimized ROS balance and the relationship between mitochondrial respiration and ROS. Biochim. Biophys. Acta.

[60] Allen J.F. (2015). Why chloroplasts and mitochondria retain their own genomes and genetic systems: colocation for redox regulation of gene expression. Proc. Natl. Acad. Sci. USA..

[61] Mailloux R.J., Harper M.E. (2011). Uncoupling proteins and the control of mitochondrial reactive oxygen species production. Free Radic. Biol. Med..

[62] Sarewicz M., Dutka M., Pintscher S., Osyczka A. (2013). Triplet state of the semiquinone-Rieske cluster as an intermediate of electronic bifurcation catalyzed by cytochrome bc1. Biochemistry.

[63] Marais A., Sinayskiy I., Petruccione F., van Grondelle R. (2015). A quantum protective mechanism in photosynthesis. Sci. Rep..

[64] Roston D., Islam Z., Kohen A. (2014). Kinetic isotope effects as a probe of hydrogen transfers to and from common enzymatic cofactors. Arch. Biochem. Biophys..

[65] Moradi N., Scholkmann F., Salari V. (2015). A study of quantum mechanical probabilities in the classical Hodgkin–Huxley model. J. Integr. Neurosci..

[66] Summhammer J., Salari V., Bernroider G. (2012). A quantum-mechanical description of ion motion within the confining potentials of voltage-gated ion channels. J. Integr. Neurosci..

[67] Skulachev V.P. (2001). Mitochondrial filaments and clusters as intracellular power-transmitting cables. Trends Biochem. Sci..

[68] Reynaud J.A., Labbe H., Lequoc K., Lequoc D., Nicolau C. (1989). Electric field-induced fusion of mitochondria. FEBS Lett.

[69] Srobar F. (2012). Frohlich systems in cellular physiology. Prag. Med. Rep..

[70] Priel A., Ramos A.J., Tuszynski J.A., Cantiello H.F. (2008). Effect of calcium on electrical energy transfer by microtubules. J. Biol. Phys..

[71] Pokorny J., Pokorny J., Foletti A., Kobilkova J., Vrba J., Vrba J. (2015). Mitochondrial dysfunction and disturbed coherence: gate to cancer. Pharmaceuticals.

[72] Arismendi-Morillo G. (2009). Electron microscopy morphology of the mitochondrial network in human cancer. Int. J. Biochem. Cell Biol..

[73] Wai T., Langer T. (2016). Mitochondrial dynamics and metabolic regulation. Trends Endocrinol. Metab..

[74] Tamulis A., Grigalavicius M. (2011). The emergence and evolution of life in a “fatty acid world” based on quantum mechanics. Orig. Life Evol. Biosph..

[75] Hameroff S., Penrose R. (2014). Consciousness in the universe: a review of the 'Orch OR' theory. Phys. Life Rev..

[76] Layfield J.P., Hammes-Schiffer S. (2014). Hydrogen tunneling in enzymes and biomimetic models. Chem. Rev..

[77] McGlynn S.E., Chadwick G.L., Kempes C.P., Orphan V.J. (2015). Single cell activity reveals direct electron transfer in methanotrophic consortia. Nature.

[78] Pfeffer C., Larsen S., Song J., Dong M., Besenbacher F., Meyer R.L., Kjeldsen K.U., Schreiber L., Gorby Y.A., El-Naggar M.Y. (2012). Filamentous bacteria transport electrons over centimetre distances. Nature.

[79] Wegener G., Krukenberg V., Riedel D., Tegetmeyer H.E., Boetius A. (2015). Intercellular wiring enables electron transfer between methanotrophic archaea and bacteria. Nature.

[80] Dudkina N.V., Folea I.M., Boekema E.J. (2015). Towards structural and functional characterization of photosynthetic and mitochondrial supercomplexes. Micron.

[81] Lapuente-Brun E., Moreno-Loshuertos R., Acin-Perez R., Latorre-Pellicer A., Colas C., Balsa E., Perales-Clemente E., Quiros P.M., Calvo E., Rodriguez-Hernandez M.A. (2013). Supercomplex assembly determines electron flux in the mitochondrial electron transport chain. Science.

[82] Melo A.N.P., Teixeira M. (2016). Supramolecular organization of bacterial aerobic respiratoy chains: from cells and back. Biochim. Biophys. Acta.

[83] Tyner K.M., Kopelman R., Philbert M.A. (2007). “Nanosized voltmeter” enables cellular-wide electric field mapping. Biophys. J..

[84] Jandova A., Pokorny J., Pokorny J., Kobilkova J., Nedbalova M., Cocek A., Jelinek F., Vrba J., Vrba J., Dohnalova A. (2015). Diseases caused by defects of energy level and loss of coherence in living cells. Electromagn. Biol. Med..

[85] Fisher M.P.A. (2015). Quantum cognition: the possibility of processing with nuclear spins in the brain. Ann. Phys..

[86] Michel D. (2013). Life is a self-organizing machine driven by the informational cycle of Brillouin. Orig. Life Evol. Biosph..

[87] Le Bourg E. (2012). Forecasting continuously increasing life expectancy: what implications?. Ageing Res. Rev..

[88] Robertson H.T., Allison D.B. (2012). A novel generalized normal distribution for human longevity and other negatively skewed data. PloS One.

[89] Weon B.M. (2015). A solution to debates over the behavior of mortality at old ages. Biogerontology.

[90] Kaeberlein M., Rabinovitch P.S., Martin G.M. (2015). Healthy aging: the ultimate preventative medicine. Science.

[91] Lane N. (2003). A unifying view of ageing and disease: the double-agent theory. J. Theor. Biol..

[92] Salminen A., Huuskonen J., Ojala J., Kauppinen A., Kaarniranta K., Suuronen T. (2008). Activation of innate immunity system during aging: NF-kB signaling is the molecular culprit of inflamm-aging. Ageing Res. Rev..

[93] Franceschi C., Bonafe M., Valensin S., Olivieri F., De Luca M., Ottaviani E., De Benedictis G. (2000). Inflamm-aging. An evolutionary perspective on immunosenescence. Ann. N.Y. Acad. Sci..

[94] Speakman J.R., Mitchell S.E. (2011). Caloric restriction. Mol. Aspects Med..

[95] Adler M.I., Bonduriansky R. (2014). Why do the well-fed appear to die young? A new evolutionary hypothesis for the effect of dietary restriction on lifespan. Bioessays.

[96] Baker D.J., Childs B.G., Durik M., Wijers M.E., Sieben C.J., Zhong J., Saltness R.A., Jeganathan K.B., Verzosa G.C., Pezeshki A. (2016). Naturally occurring p16(Ink4a)-positive cells shorten healthy lifespan. Nature.

[97] Lowe D., Horvath S., Raj K. (2016). Epigenetic clock analyses of cellular senescence and ageing. Oncotarget.

[98] de Magalhaes J.P. (2012). Programmatic features of aging originating in development: aging mechanisms beyond molecular damage?. FASEB J..

[99] Wallace D.C., Fan W. (2010). Energetics, epigenetics, mitochondrial genetics. Mitochondrion.

[100] Salminen A., Kaarniranta K., Hiltunen M., Kauppinen A. (2014). Krebs cycle dysfunction shapes epigenetic landscape of chromatin: novel insights into mitochondrial regulation of aging process. Cell. Signal..

[101] West A.P., Shadel G.S., Ghosh S. (2011). Mitochondria in innate immune responses. Nat. Rev. Immunol..

[102] Barja G. (2013). Updating the mitochondrial free radical theory of aging: an integrated view, key aspects, and confounding concepts. Antioxid. Redox Signal..

[103] Tower J. (2015). Mitochondrial maintenance failure in aging and role of sexual dimorphism. Arch. Biochem. Biophys..

[104] Li M., Schroder R., Ni S., Madea B., Stoneking M. (2015). Extensive tissue-related and allele-related mtDNA heteroplasmy suggests positive selection for somatic mutations. Proc. Natl. Acad. Sci. U.S.A..

[105] Salomon J.A., Wang H., Freeman M.K., Vos T., Flaxman A.D., Lopez A.D., Murray C.J. (2012). Healthy life expectancy for 187 countries, 1990–2010: a systematic analysis for the Global Burden Disease Study 2010. Lancet.

[106] Tapia P.C. (2006). Sublethal mitochondrial stress with an attendant stoichiometric augmentation of reactive oxygen species may precipitate many of the beneficial alterations in cellular physiology produced by caloric restriction, intermittent fasting, exercise and dietary phytonutrients: "Mitohormesis" for health and vitality. Med. Hypotheses.

[107] Allocati N., Masulli M., Di Ilio C., De Laurenzi V. (2015). Die for the community: an overview of programmed cell death in bacteria. Cell Death Dis.

[108] Marraffini L.A. (2015). CRISPR-Cas immunity in prokaryotes. Nature.

[109] Heussler G.E., Cady K.C., Koeppen K., Bhuju S., Stanton B.A., O'Toole G.A. (2015). Clustered regularly interspaced short palindromic repeat-dependent, biofilm-specific death of *Pseudomonas aeruginosa* mediated by increased expression of phage-related genes. mBio.

